# Pharmacogenomic study on anti-VEGF medicine in treatment of macular Neovascular diseases: a study protocol for a prospective observational study

**DOI:** 10.1186/s12886-018-0812-4

**Published:** 2018-07-24

**Authors:** Jin Jing, Shen Yinchen, Chen Xia, Wang Jing, Chen Chong, Xu Xun, Huang Hengye, Liu Kun

**Affiliations:** 1 Department of Ophthalmology, Shanghai General Hospital, Shanghai Jiao Tong University School of Medicine, Shanghai, 200080 People’s Republic of China; 2 Shanghai Key Laboratory of Ocular Fundus Diseases, Shanghai, 200080 People’s Republic of China; 3 Shanghai Engineering Center for Visual Science and Photomedicine, Shanghai, 200080 People’s Republic of China; 4 School of Public Health, Shanghai Jiao Tong University School of Medicine, Shanghai, 200025 People’s Republic of China

**Keywords:** Anti-VEGF, Macular neovascular diseases, Conbercept, Pharmacogenomic

## Abstract

**Background:**

Macular neovascular diseases can cause severe vision loss. A newly approved anti—VEGF drug Conbercept has shown good efficacy and safety in rigorous random controlled trials (RCT), however, it cannot fully reflect the clinical application of Conbercept in real world clinical practice. Moreover, anti-VEGF drugs are expensive and often require multiple treatments, and some patients have poor or even no response to the drugs,this resulted enormous waste of medical resources. Therefore, how to find out those patients who have good response, and how to develop individualized therapeutic regimen in real world need to be urgently investigated in the aspect of pharmacogenomics and pharmacometabolomics.

**Methods:**

This study is a multicenter, prospective, observational study of Conbecept treating macular neovascular diseases in China. Patients suffered from age-related macular degeneration, polypoidal choroidal vasculopathy, and pathological myopia who already planned to receive Conbercept treatment will be recruited. We aimed to enroll more than 5000 patients from 43 ophthalmic centers in China. Patients’ clinical data and blood samples will be collected during the one-year follow-up period. Finally, the safety and efficacy of Conbercept, and the potential predictors of patients’ response to Conbercept will be investigated by pharmacogenomics and pharmacometabolomics analysis.

**Discussion:**

This study will provide important data of Conbercept in treating macular neovascular diseases in real world. Besides, finding the predictor of patients’ response will help doctor make more precise individualized therapeutic regimens.

**Trial registration:**

ClinicalTrials.gov, NCT03128463. Registered on 9 March 2017.

**Electronic supplementary material:**

The online version of this article (10.1186/s12886-018-0812-4) contains supplementary material, which is available to authorized users.

## Background

Macular neovascular disease characterized by subfoveal choroidal neovascularization (CNV) remains the leading cause of irreversible blindness that impacts quality of life [1–4] including age-related macular degeneration (AMD) [5], polypoidal choroidal vasculopathy (PCV) [6], pathological myopia (PM) [7, 8] and so on. Previous therapies such as photodynamic therapy with Verteporfin showed limited success [9]. Due to the similarities in pathogenesis of CNV, anti-vascular endothelial growth factor therapy (anti-VEGF) has been shown to be effective and has become the first choice for the treatment of CNV [10].

Conbercept (Lumit®, Chengdu Kanghong Biotech Co., Ltd., P. R. China) is an anti -VEGF drug developed in China in recent years. It has been approved by the China Food and Drug Regulatory Administration (CFDA) for the treatment of neovascular AMD and was recently admitted directly to the Phase III clinical trials in the U.S. Food and Drug Administration (FDA). This drug competitively prevents the binding of VEGF to its receptor and inhibits the downstream pathway activation, and has a higher binding affinity to the most potent pro-angiogenic alternative splicing of VEGF——VEGFA than other widely used anti-VEGF drugs [11, 12].

In our previous phase I and II studies, the intravitreal administration of Conbercept successfully improved visual acuity and reduced central retinal thickness and CNV area in patients with neovascular AMD. AURORA study and its subgroup analysis showed that Conbercept has good efficacy and safety in treating macular neovascular diseases such as PCV and AMD [13, 14]. After three months’ injection, average changes in BCVA in the 0.5- and 2.0-mg groups were 8.97 and 10.43 letters, respectively [14]. During the 12 months, patients have good tolerances for the repeated intravitreal injections of Conbercept [14].

Although the above clinical trial results showed that Conbercept had good efficacy and safety in treating AMD [14], the exact therapeutic regimen and the efficacy still needs to be further studied in real world. The reasons are as follows: i) Limitation of external validity of randomized controlled trials (RCTs) conducted in AMD, because they may not fully reflect the clinical application of Conbercept in real world; ii) Lack of RCT of Conbercept in the treatment of CNV secondary to PM, and the efficacy of Conbercept in PCV only resulted from subgroup analysis. Therefore, further clinical trials of Conbercept in other macular neovascular disease need to be explored, and our study may provide the preliminary evidence for further RCTs in PM and PCV; iii) Anti-VEGF therapy poses significant financial and treatment burden on patients and social insurance system due to frequent injections and visits. Moreover, some patients have poor or even no response to the drugs. It is important to find out those who have good response and develop individualized therapeutic regimen with the use of pharmacogenomics and pharmacometabolomics technologies.

Therefore, we present the design and methodology of a prospective study to investigate the efficacy and safety data of Conbercept in treating macular neovascular disease in clinical practice and explore the relationships of phenotype and the effectiveness of the Conbercept through pharmogenomics and pharmacometabolomics in China.

## Methods and analysis

### Study design and objectives

We intended to conduct a prospective observational study within multicenter to investigate the efficacy and safety of Conbercept in real world and explore the potential predictors of patients’ response to Conbercept (Protocol no. 2.0 date 20 October 2017).

This program starts from March 2017 (FPFV, First Patient First Visit) and will finish in December 2018 (LPLV, Last Patient Last Visit). We recruit eligible participants attending 43 ophthalmologic centers in China (The case report form is attached in Additional file 1). Patients are screened according to unified diagnostic standard guideline or consensus of Chinese Ocular Fundus Disease Society. Those who subject to all of criteria, we will explain our study to patients and their families. Finally, consent form will be signed if they are willing to participate in our study. Their clinical data will be collected during the five visits: baseline (V1: enrollment period, first Conbercept treatment), following 1 month (V2), following 3 month (V3), following 6 month (V4), following 12 month (V5) during the 1-year follow-up. (The clinical information collected during each visit is attached in Additional file 2) Patients whose actual visits are within 7 days before or after the registration time can be considered available. If the participants come back for visit but their visit time is beyond the time window or failed to register the clinic follow-up for some reason, the researchers should implement a registration by phone call. If the participants cannot be contacted by telephone, the visit can be considered lost. All the reasons and processes should be documented. The case report form is attached (Additional file 1).

**Fig. 1:**
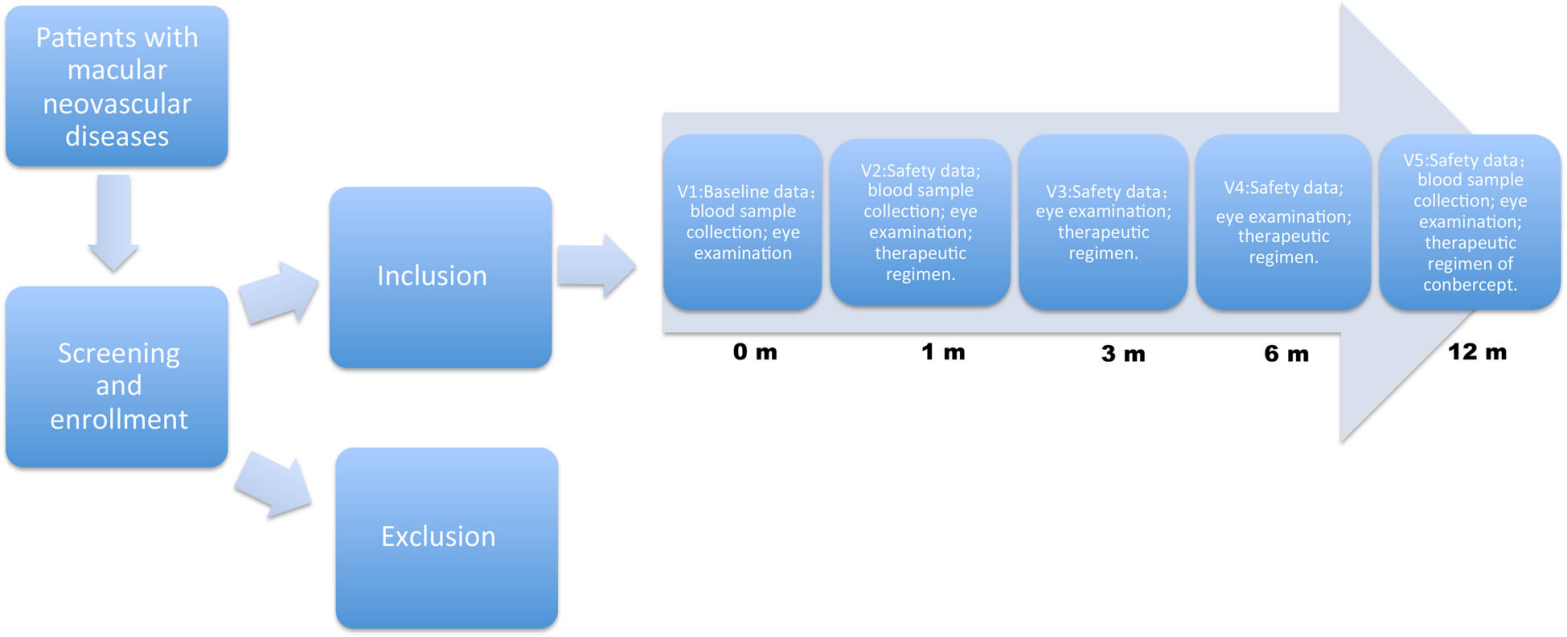
Flowchart of recruitment and observation procedures of this study. 1 m, 3 m,6 m, and 12 m means 1 month, 3 months, 6 months, and 12 months after Conbercept treatment

Among subjects, 500 patients will be selected to conduct pharmacometabolomics analysis compared with 500 healthy volunteers for detecting disease-associated metabolites. After the evaluation of patients’ curative parameters (listed below), preliminary pharmacogenomics and pharmacometabolomics analysis will be carried out. We will pick up 500 patients, half of them have good response from significant effective group and half have no response from deterioration group, their blood samples will be analyzed by pharmacogenomics and pharmacometabolomics to find out response-associated predictors, and then the predictors of patients’ response to Conbercept will be further validated in all of the recruited subjects. Figure 1 is the study design flow.

### Participants and recruitment

More than 5000 patients will be recruited from 43 ophthalmic centers in china that suffer from AMD, PCV, and PM (List of Ophthalmic Center in Additional file 3). And it is the first time for them to receive ocular injection of Conbercept. Doctors in outpatient clinic who diagnose and prescribe are independent of the investigators in this study. The 43 ophthalmic centers cover most regions of China. The recruitment capacity of each center will be mainly depended on the daily outpatient and registration capacity, as well as follow-up conditions. Participation is voluntary and consent is obtained before the procedures. Until now, the recruitment and primary pharmacogenomic analysis is ongoing and 3055 patients have been recruited.

### Inclusion criteria:

1. Age ≥ 18 years;
2. Patients are diagnosed as AMD, PCV and CNV secondary to PM;
3. Patients will receive intravitreal injection of Conbercept;
4. Patients are willing to have a long-term follow–up in the clinical center;
5. Signed consent form.

### Exclusion criteria:

1. Participate in other interventional therapy at the same time;
2. Received anti-VEGF treatment (including intravitreal injection or systematic application) within three months prior to enrollment.

### Data collection and management

Investigators in the 43 ophthalmic centers should follow Standard Operating Procedures (SOPs) during clinical data and blood sample collection, data recording and transfer, sample storing and transfer. All of the data will be filled in the electronic case report form (CRF). At the initial visit (V1), information about the participant’s sex, age, body weight, height, vital signs, medical/surgical history, concomitant medications and eye conditions will be recorded. For ETDRS vision, eye examinations, optical coherence tomography (OCT), fluorescein fundus angiography (FFA) will be checked by clinicians who have got international unified certification. The eye conditions, treatment records, accompanying medications and adverse events are recorded by practiced clinicians at five visits. Clinical images will be uniformly transferred to Shanghai Jiaotong University Ophthalmic Reading Center and evaluated by qualified graders. The specific items are listed in Table 1.

**Table 1:** Assessment protocol for the current study from V1 to V5

Visit of data entry	V1	V2	V3	V4	V5
Meets enrollment / exclusion criteria	X				
Month		1	3	6	12
Days		30 (± 7) days after first injection	90 (± 7) days after first injection	180 (± 7) days after first injection	360 (± 7) days after first injection
Consent form	X				
Demographic data	X				
Systemic and ocular disease history	X				
Best corrected visual acuity	X	X	X	X	X
Optical coherence tomography	X	X	X	X	X
Accompanying medication	X	X	X	X	X
Data record	X	X	X	X	X
Treatment program records		X	X	X	X
Adverse events	X	X	X	X	X

The clinical data and fundus imaging data will be uploaded to Electronic Data Capture (EDC) System called Oracle Clinical and Oracle Remote Data Capture (OC/RDC) churned out according SOPs. The EDC system has been checked by third party and meets international standard. And data from different centers will be managed together and go through logistic check. Each modification trace can be recorded to make sure the data is real and reliable.

Patients’ blood samples will be collected in coagulation-promoting tubes (8 ml) and EDTA procoagulant tubes (8 ml), and then be centrifuged for 20 min at 4000 rpm to separate serum and plasma respectively. All the samples will be send to Ophthalmic Biobank of Shanghai Jiaotong University via cold-chain logistic and then stored at − 80 °C.

### Main outcome measures

#### Primary outcome

The primary outcome is visual changes primarily recorded as best-corrected visual acuity (BCVA) in EDTRS letters at V2, V3, V4, and V5 minus baseline vision (V1). The efficacy was graded as significantly effective (visual improvement ≥15 letters in EDTRS); effective (visual improvement ≥5 letters and < 15 letters in EDTRS); invalid (visual improvement < 5 letters and visual reduction< 5 letters in EDTRS); deterioration (visual reduction≥5 letters in EDTRS) [15]. Moreover, patients graded as significantly effective and effective that is BCVA improvement≥5 letters in EDTRS are defined as having response to Conbercept treatment and the response rate (the rate of patients graded as significantly effective and effective) will be assessed in the whole study population and in each diseases.

#### Secondary outcome

1. The changes of retinal edema will be evaluated by central fovea retinal thickness (CRT in μm) at V2, V3, V4 and V5.
2. The different therapeutic regimens of Conbercept prescribed by doctors and applied on different diseases in real-world clinical practice will be assessed, including number of treatments, frequency of injection (times per year), interval time (months).
3. Response-related factors for Conbercept to macular neovascular diseases after 3 months’ treatment will be examined. We will compare the macular leakage area (mm^2^) and the CNV area (mm^2^) to baseline in FFA between patients who response to Conbercept treatment and patients with no response, and their relationship with the responders and nonreponders will be further analyzed.
4. The potential biomarker of patients’ response to Conbercept will be investigated by pharmacogenomics and pharmacometabolomics as mentioned above in study design. The genomic variation between patients who respond to Conbercept treatment and patients who have no response to Conbercept drug treatment will be detected. Conbercept-sensitive associated Single Nucleotide Variations (SNVs) will be picked out by comparing the data of single nucleotide polymorphisms dbSNP and hapMap Database to remove common single nucleotide polymorphisms (SNPs) or SNVs information. Then we will design gene panel based on Next Generation Sequencing to validate the Conbercept-sensitive genomic regions among the whole study population. Besides, prediction model will establish to figure out Conbercept-sensitive genotype in macular neovascular diseases. For pharmacometabolomics studies, blood samples of patients and healthy volunteers will be analyzed by mass spectrometry to primarily screen disease-associated metabolites. The associations of genotype and metabolites with clinical endpoint will be assessed and further validated in the whole study population. Those signatures (gene/ metabolites) will be considered to be predictors of patients who will possibly have good response to Conbercept treatment, and those in non-responders will be recognized as potential predictors of patients who do not respond to Conbercept. Finally, we will build an Oracle database about macular neovascular diseases based on clinical information, pharmacogenomics, and pharmacometabolomics.
5. Safety data: adverse events (AE) and severe adverse events (SAE) were defined according to other large-scale clinical trial on intravitreal injection of anti-VEGF drugs [14–16]. AE and SAE need to be recorded, and their relevance to drug or procedures will be estimated. The number and percentage of AE and SAE in the whole study population and in each disease will be calculated.

### Data analysis

All data will undergo statistical tests, analysis will be based on baseline and follow-up data. In case of missing value, statistician will take out corresponding countermeasures in according to the provenance mechanisms of the missing data and the practical situation. Quantitative data will be described by “mean ± standard deviation” or “Median (Range)” and compared using Student’s t test, analysis of variance (ANOVA) and nonparametric test between two or multiple groups. Data for repeated measurements will be compared using multivariate analysis of variance (MANOVA) or trend test. Qualitative data is described by “Frequency (percentage)” and analyzed by the chi-square test (or Fisher’s exact test, if appropriate), and nonparametric test in differentiation among the groups. Pearson correlation or Spearman rank correlation will be used for correlation analysis. Multivariate statistical analysis including multiple linear regression, logistic regression, cox proportional hazards model, factor analysis, path analysis will be implemented in multivariate analysis or modeling process according to the data type. In case of multiple dimensional correcting issues of confounding factors, which can not be adjusted by multivariate model, then propensity score matching (PSM) or inverse probability weighting (IPW) will be applied. Such as Nomogram or classification and regression tree (CART) will be implemented to establish predictive models which could predict patients’ response to drugs (curative effects) in different diseases and proper subgroup analysis in clinically important indicators will be applied to find out high-benefit population. The models will be evaluated using the receiver operating characteristic-derived area under the curve (AUC) or Harrell concordance index (C-index), calibration plot, and decision-curve analyses (DCAs).

For Pharmacogenomics and pharmacometabolomics, two kinds of statistical analysis will be implemented: 1) multivariate statistical analysis such as random forest, support vector machine learning, principal component analysis (PCA), partial least square discriminant analysis (PLS-DA), orthogonal partial least square discriminant analysis (OPLS-DA). 2) univariate statistical analysis, correlation analysis, and the regression analysis will be used. The predictor-ranking model will be determined by significant cutoff values (*p* < 0.05). All of data will be analyzed by R language (http://cran.r-project.org/) or SPSS (version 19.0) or SAS (University Edition) software.

To enhance comparability between outcome measures, we will select some comparable subgroups for analysis. Subgroups analysis includes comparison between healthy controls (from Ophthalmic Bio-bank of Shanghai Jiaotong University) and AMD patients, PCV patients, and CNV secondary to PM patients respectively for detecting the disease biomarkers; comparison between AMD, PCV and CNV secondary to PM patients to analyze the different signatures within different macular neovascular diseases; comparision between patients who have good response and who have no response to Conbercept in AMD patients, PCV patients, CNV secondary to PM patients respectively to explore the potential predictors of patients’ response to Conbercept treatment.

### Quality control and management

We have made SOPs, videos and OC/RDC system to train all staffs involved in this study how to collect data, and deal with blood sample. The apparatus applied to our study have been certificated by CFDA. All prescribed drugs are from hospital pharmacy, and the empty bottle of Conbercept after injection will be retrieved to ensure the dosage used.

Data Management: Clinical information should be timely, accurately and completely recorded and entered into the CRF on national network of EDC, maintaining confidentiality in accordance with the local law for data protection. The electronic CRF should be consistent with the original medical records. All adverse events, concomitant medications should be documented, serious adverse events should be reported to the relevant authorities within 24 h.

Follow-up management: Follow-up should be conducted according to required time point under the rules. The investigators should enroll in local patients or patients who plan to have regular and long-term follow-up in the hospital, and the rate of lost to follow-up should be controlled below 20%. These procedures would help to minimize possible bias.

Center management: All researchers will be checked for the qualification, and then be trained to make sure they are familiar with the study protocol and will carry out the study strictly. Each center should appoint a clinical research coordinator (CRC) for quality control, making sure the CRF form timely recorded, assisting the check and inspection, drug reception, storage, distribution and checking procedures. CRC should report to PI when they found abnormal data or non-compliances to the protocol.

## Discussion

A variety of anti-VEGF drugs for macular neovascular disease such as ranibizumab [17, 18] bevacizumab [19], aflibercept [20], and Conbercept [21] have emerged in recent years. And Conbercept has aroused wide attention for its promising capacity. In the present study, we plan to collect clinical data and blood samples from 5000 patients who suffer from macular neovascular diseases and will receive intravitreal injections of Conbercept. This prospective study, to our knowledge, has not been proposed previously in literature.

Previous RCT studies revealed that intravitreal administration of Conbercept in AMD patients leads to significant gains in BCVA, reduction in CRT, and a decrease in CNV areas [14]. However, the rigid RCT data could not fully reflect the clinical practice for its inclusion and exclusion criteria was too strict which may lead to poor external authenticity. Our study will observe and analyze data collected from clinical centers, the outcomes could be potent supplements for Conbercept applications in AMD in a larger population.

Although macular neovascular AMD is the only approved indication for Conbercept, it has shown promising potentials in its “off-label use”. Ophthalmologists have found Conbercept could improve visual acuity of other macular neovascular diseases such as PCV and CNV secondary to PM in clinical practice, and these “off-label use” have already been widely implemented. In the subgroup analysis of Aurora study, clinical data from 53 patients with PCV were retrospectively evaluated, and it showed that Conbercept could significantly improve anatomical outcomes and visual acuity [15]. Except for the above study, there is hardly any large-sample prospective study related to the “off-label use” of Conbercept in macular neovasular diseases such as PCV and CNV secondary to PM. Recently, the guidance and consensus statement on CNV secondary to PM has been carried out by experts from many countries, and anti-VEGF drugs have been considered as first-line therapy for myopic CNV [21]. Therefore, this prospective study may provide updated high–level evidence for Conbercept treatment in PCV and CNV secondary to PM.

Other anti-VEGF drugs such as ranibizumab or bevacizumab have been studied in treating PCV and CNV secondary to PM, and they had demonstrated effects on improving visual acuity and CRT [21–23]. However, the mechanisms of Conbercept in inhibiting VEGF function is different from the other anti-VEGF drugs, and whether the newly-developed drug is safe and efficacious for PCV and CNV secondary to PM have not been estimated. In our study, numerous clinical data we collected may provide convincing evidence for applications of Conbercept in PCV and CNV secondary to PMand may benefit further RCTs.

In the present study, we will use pharmacogenomics and pharmacometabolomics to investigate the biomarkers on the efficacy of Conbercept treatment. It has been reported that some patients have poor or no response to anti-VEGF drugs [24–26], and extensive and repeated administration of anti-VEGF drugs often leads to unnecessary wastes. Previously, researchers had found that gene polymorphism is associated with AMD patients’ response to intravitreal injection of anti-VEGF drugs and therapeutic regimen [27]. However, most of the studies analyzed assessed limited number of SNPs and their interactions with patients’ response to ranibizumab, the number of injections needed to maintain visual gains [27]. In this study, whole genome information may provide much more important data. Besides, there are no reported study involving pharmacometabolomics on Conbercept treating AMD, PCV, and CNV secondary to PM. In our study, we will select some patients for whole-genome sequencing and pharmacometabolomics analysis, then validate among larger populations. We expect to find new predictors of patients’ response to Conbercept, and the outcome would help doctors to treat these patients effectively, further develop individualized therapy, and improve patients’ compliance.

Although we will comprehensively investigate the clinical data, genome information, and metabolic factors of Chinese population, the current study still lacks clinical information in other human races. Therefore, further worldwide clinical studies are still needed to testify the above-mentioned results in other human races.

## Additional files

Additional file 1: Case report form. (PDF 114 kb)

Additional file 2: Clinical information collected from V1 to V5. (PDF 55 kb)

Additional file 3: Ophthalmic centers. (DOCX 18 kb)

## Abbreviations

AMD: Age-related Macular Degeneration
BCVA: best-corrected visual acuity
CNV: Choroidal Neovascularization
PCV: Polypoidal Choroidal Vasculopathy
PM: Pathological Myopia
SNPs: Single Nucleotide Polymorphisms
SNVs: Single Nucleotide Variations
VEGF: Vascular Endothelial Growth Factor
