# Supplementary material for: Pharmacogenomic study on anti-VEGF medicine in treatment of macular Neovascular diseases: a study protocol for a prospective observational study
Source: BMC Ophthalmol. 2018 Jul 24;18:181. doi: 10.1186/s12886-018-0812-4 (PMC6057085; doi:10.1186/s12886-018-0812-4)
Supplement: Supplementary file 1 — Case report form. (PDF 114 kb) [file 12886_2018_812_MOESM1_ESM.pdf]

A prospective, observational, multicenter and register study on conbercept treating macular neovascular diseases

Case report form

Version 1.0

2016． 11． 05

A prospective, observational, multicenter and register study on conbercept treating macular neovascular diseases

Medical record
version 1.0

Patient's name: ____________________

Patient's number： ________________

Research center： _________________

Organizer：Shanghai General Hospital

Index

1、 This medical record should be written by investigators or authorized person.

2、 Medical record and patients' number should be filled in after informed consent is obtained, and patients' name should be consistent with ID card.

3、 This record should be written timely, accurately, completely，and can not be leaved out and amended freely. The data can not be faked.

Notifications for filling the records：

1) This record should be written in black or black blue pen；
2) This record should be kept intact, and pages can not be missing or replaced；
3) Please write"×" in "□"；
4) If there are mistakes needs to be corrected, it can't be blacked; the mistakes should be crossed with a single line, and the corrections, signatures, and date should be written besides.
5) Investigators should sign in full name clearly， and the data should be recorded in 8 digits, such as: 2010/07/20.

Informed consent

Signed informed？ □Yes □No

Date：201__ /__ __ /__ __

Whether the patient got the copy of informed consent？

□Yes □No

Investigator(signature)： ____________________________

V1： Baseline

Visit date：201__ /__ __ /__ __

Basic information

Name：__________ Gender：□Male □Female

Birth date： ________year_______month_______date

Ethnic： ________ Educational level： __________

Profession： ______________ Telephone： _______

Home address： ______________________________________

ID number： □□□□□□□□□□□□□□□□□□

Inclusion and exclusion criteria

**Inclusion criteria**	Yes	No
Signed informed	□	□
Patients were diagnosed with macular neovascular diseases (age-related macular degeneration (AMD), polypoidal choroidal vasculopathy (PCV), choroidal neovascularization secondary to pathological myopia (PM)); no gender requirement; age ≥ 18year.	□	□

Patients will receive intravitreal injection of conbercept	□	□
Patients are willing to have a long-term follow–up in the clinical center.	□	□

Exclusion criteria	Yes	No
Participate in other intervention therapy at the same time	□	□
Received anti-VEGF treatment (including intravitreal injection or systematic application) within three months prior to enrollment.	□	□

General conditions

Vital signs：T____°C; P____ beats/min;

R____ times/min; BP__/__mmHg.

History of systematic diseases

（Cardiovascular events: myocardial infarction, stroke, thromboembolic events; obesity,

coronary artery disease Disease, family history,

high cholesterol, hyperlipidemia, hypertension,

diabetes and other medical history）

Concomitant medication /important Non-drug treatments_

Targeted eye：□OD □OS

Blood sample collection： □Yes □No

Eye examination：

ETDRS vision：OD______ OS_______

Intraocular pressure（mmHg）：___________

____

OD______ OS______

Cornea: ____________________________________

Anterior chamber: ___________________________

Iris: ____

Lens: _______________________________________

Vitreous： ________________________________

Fundus: _____________________________________

Imaging examinations：

Optical coherence coherence tomography(OCT)：

OCT central foveal retinal thickness： _____μm

OCT volume of central fovea： ___________mm^3

Fluorescein fundus angiography（FFA）：

CNV area： ____ ____.____ ____ mm^2；

Macular leakage area： ____ ____.____ ____ mm^2；

Focus area： ____ ____.____ ____ mm^2

Injection date：201__ /__ __ /__ __

Investigator(signature)： ________________

Date： ________________

V2：1 month after first injection

Visit date：201__ /__ __ /__ __

Last injection date： 201__ /__ __ /__ __

Targted eye：□OD □OS

Vital signs：T____°C; P____ beats/min;

R____ times/min; BP__/__mmHg.

Concomitant medication /important Non-drug treatment_

____

____

____

Blood sample collection： □Yes □No

Adverse events（last visit till now）

Adverse events □None □Yes→If any，please record in detail (including adverse event name, part of the body, start time, end time, severity, measures taken, the outcome of adverse events, correlation with the study treatment, etc.）

____

____

____

____

Eye examination：

ETDRS vision：OD______ OS_______

Intraocular pressure（mmHg）：___________

OD______ OS_______

Cornea: _____________________________________

Anterior chamber: ___________________________

Iris: ____

Lens: _______________________________________

Vitreous： __________________________________

Fundus: _____________________________________

Imaging examinations：

Optical coherence coherence tomography(OCT)：

OCT central foveal retinal thickness ： _____μm

OCT volume of central fovea： ___________mm^3

Fluorescein fundus angiography（FFA）：

CNV area： ____ ____.____ ____ mm^2；

____

Macular leakage area： ____ ____.____ ____ mm^2；

Focus area： ____ ____.____ ____ mm^2

Injection date：201__ /__ __ /__ __

Investigator(signature)： ________________

Date： ________________

V3：3 months after first injection

Visit date：201__ /__ __ /__ __

Targted eye：□OD □OS

Vital signs：T____°C;   P____ beats/min;

R____ times/min;   BP__/__mmHg.

Concomitant medication /important Non-drug treatment_

____

____

____

Adverse events   □None   □Yes→If any，please record in detail (including adverse event name, part of the body, start time, end time, severity, measures

taken, the outcome of adverse events, correlation with the study treatment, etc.）

____

____

____

Eye conditions

Records of conbercept treatment (including date of treatment and number of injections; treatment status: under treatment, treatment suspended ,quit, the reasons ; regimen record ： left / right eye, specific treatment registration)

____

____

Eye examination：

ETDRS vision：OD______ OS_______

Intraocular pressure（mmHg）： ___________

OD______ OS_______

Cornea: _____________________________________

Anterior chamber: ___________________________

Iris: ____

Lens: ____

Vitreous： ___________________________________

Fundus: ______________________________________

Imaging examinations:

Optical coherence coherence tomography(OCT)：

OCT central foveal retinal thickness： _____μm

OCT volume of central fovea： ___________mm^3

Fluorescein fundus angiography（FFA）：

CNV area： ____ ____.____ ____ mm^2；

Macular leakage area： ____ ____.____ ____ mm^2；

Focus area： ____ ____.____ ____ mm^2

Injection date： 201__ /__ __ /__ __

Investigator(signature)： ________________

Date： ________________

V4： 6 months after first injection

Visit date： 201__ /__ __ /__ __

Targted eye： □OD □OS

Vital signs：T____°C; P____ beats/min;

R____ times/min; BP__/__mmHg.

Concomitant medication /important Non-drug treatment_

Adverse events □None □Yes→If any，please record in detail (including adverse event name, part of the body, start time, end time, severity, measures taken, the outcome of adverse events, correlation with the study treatment, etc.）

Eye conditions

Records of conbercept treatment (including date of treatment and number of injections; treatment status: under treatment, treatment suspended ,quit, the reasons ; regimen record ： left / right eye, specific treatment registration)

____

____

Eye examination：

ETDRS vision：OD______ OS_______

Intraocular pressure（mmHg）：___________

OD______ OS_______

Cornea: _____________________________________

Anterior chamber: ___________________________

Iris: ____

Lens: _______________________________________

Vitreous： __________________________________

Fundus: _____________________________________

Imaging examinations：

Optical coherence coherence tomography(OCT)：

OCT central foveal retinal thickness： _____μm

OCT volume of central fovea： ___________mm^3

Fluorescein fundus angiography（FFA）：

CNV area： ____ ____.____ ____ mm^2;

Macular leakage area： ____ ____.____ ____ mm^2;

Focus area： ____ ____.____ ____ mm^2

Injection date：201__ /__ __ /__ __

Investigator(signature)：________________

Date：________________

V5：12 months after first injection

Visit date：201__ /__ __ /__ __

Targted eye：□OD □OS

Vital signs：T____°C; P____ beats/min;

R____ times/min; BP__/__mmHg.

Concomitant medication /important Non-drug treatment_

____

____

____

Blood sample collection： □Yes □No

Adverse events（last visit till now）

Adverse events □None □Yes→If any，

please record in detail (including adverse event name,

part of the body, start time, end time, severity, measures taken, the outcome of adverse events, correlation with the study treatment, etc.）

____

____

____

____

Eye conditions

Record of conbercept treatment（including date of treatment and number of injections; treatment status: under treatment, treatment suspended ,quit, the reasons ; regimen record ： left / right eye, specific treatment registration)

____

____

Eye examination：

ETDRS vision：OD______ OS_______

Intraocular pressure（mmHg）： ___________

OD______ OS_______

Cornea: ______________________________________

Anterior chamber: ____________________________

Iris: ____

Lens: ____

Vitreous： ___________________________________

Fundus: ______________________________________

Imaging examinations：

Optical coherence coherence tomography(OCT)：

OCT central foveal retinal thickness ： _____μm

OCT volume of central fovea： ___________mm^3

Fluorescein fundus angiography（FFA）：

CNV area： ____ ____.____ ____ mm^2；

Macular leakage area： ____ ____.____ ____ mm^2；

Focus area： ____ ____.____ ____ mm^2

Injection date：201__ /__ __ /__ __

Investigator(signature)： ________________

Date： ________________
