# Supplementary material for: Pharmacogenomic study on anti-VEGF medicine in treatment of macular Neovascular diseases: a study protocol for a prospective observational study
Source: BMC Ophthalmol. 2018 Jul 24;18:181. doi: 10.1186/s12886-018-0812-4 (PMC6057085; doi:10.1186/s12886-018-0812-4)
Supplement: Supplementary file 2 — Clinical information collected from V1 to V5. (PDF 55 kb) [file 12886_2018_812_MOESM2_ESM.pdf]

Supplemental table. Clinical information collected from V1 to V5

V1（baseline）	
Demographics	Name, sex, date of birth, race, ethnicity, education level, occupation, contact details
Vital signs	Height, weight, body temperature, respiration, pulse and blood pressure
History of systemic disease	Cardiovascular events: myocardial infarction, stroke, thromboembolic events; obesity, coronary artery disease Disease, family history, high-cholesterol， hyperlipidemia， hypertension， diabetes

	and other medical history
Concomitant medication / important non-drug treatment	Any ongoing drug / therapy
Blood sample collection	Patients need to collect baseline peripheral blood (procoagulant tube and EDTA anticoagulant tubes) for further genomics and metabolomics detection.
Eye conditions	
Eye disease history	Refractive errors; eye injury;<br>Medical history (including duration;<br>Intraocular injection history and drug name;<br>Previous adverse reactions and severe adverse

	reactions, etc.)
Eye examination	1. Best corrected visual acuity (BCVA) using ETDRS visual acuity table；<br>2. Sit lamp examination<br>3. Optical coherence coherence tomography, OCT)<br>4. Intraocular pressure detection<br>5. Color fundus photography<br>6. Fluorescein fundus angiography（FFA）<br>7. Indocyanine green angiography（ ICGA） (if any)
V2 (1 month after treatment)	
Concomitant medication / important non-drug treatment	Any ongoing drug / therapy
Blood sample collection	After the first month of treatment patients' peripheral blood sample need to be collected (one procoagulant tube and one EDTA anticoagulant tube) for further genomics and metabolomics

	detection.
Eye conditions	
Conbercept therapy recording	1. Date of treatment and number of injections;<br>2. Treatment status: under treatment, treatment suspended, quit, and the reasons)<br>3. Regimen record (Left / right eye, specific treatment registration)
Eye examination	1. Best corrected visual acuity (BCVA) using ETDRS visual acuity table<br>2. Slit lamp fundus examination and optical coherence tomography (OCT)<br>3. Intraocular pressure detection (IOP)<br>4. Color fundus photography

V3 (3 months)/<br>V4 (6 months)	
Concomitant medication /<br>important non-drug treatment	Any ongoing drug / therapy
Safety	Doctors need to carefully ask the patients' adverse events / severe adverse events since participated in the registration study (including adverse event name, location, start time end time, the severity classification, the measures taken, the outcome of adverse events, the relationship with the study treatment, whether the adverse events affects eyes, etc.), and fill the follow-up table
Eye conditions	
Compressor treatment Case record	1. Date of treatment and number of injection;<br>2. Treatment status: under treatment, treatment

	suspended, quit, and the reasons)<br>3. Regimen record (Left / right eye, specific treatment registration)
Eye examination	1. Best corrected visual acuity (BCVA) using ETDRS visual acuity table;<br>2. Sit lamp examination<br>3. Optical coherence coherence tomography, OCT)<br>4. Intraocular pressure detection<br>5. Color fundus photography<br>6. Fluorescein fundus angiography（FFA）<br>7. Indocyanine green angiography（ ICGA）(if any)
V5 (12 months)	
Concomitant medication / important Non-drug treatment	Any ongoing drug / therapy
Blood sample collection	After 12 months of treatment, patients' peripheral

	blood sample need to be collected (one procoagulant tube and one EDTA anticoagulant tube) for further genomics and metabolomics detection
Safety	Doctors need to carefully ask patients' adverse events since participation /severe adverse events (including adverse event name, part of the body, start time, end time, severity, measures taken, the outcome of adverse events, correlation with the study treatment, etc.), and fill up follow-up table
Eye conditions	
Conbercept treatment	Date of treatment<br>Number of intravitreal injection
Case record	1. Date of treatment and number of injection;<br>2. Treatment status: under treatment, treatment

	suspended, quit, and the reasons)<br><br>3. Regimen record (Left / right eye, specific treatment registration)
Eye examination	1. Best corrected visual acuity (BCVA) using ETDRS visual acuity table;<br><br>2. Slit lamp fundus examination;<br><br>3. Optical coherence tomography (OCT);<br><br>4. Intraocular pressure detection (IOP);<br><br>5. Color fundus photography.
Note	1. If patients do not agree to blood sampling or researchers in practice did not collect blood samples, does not affect the patient's enrollment in this registration study.<br><br>2. If the patient fails to come to the clinic, then the

	researchers should make a phone call follow up.
