# Supplementary material for: Pharmacogenomic study on anti-VEGF medicine in treatment of macular Neovascular diseases: a study protocol for a prospective observational study
Source: BMC Ophthalmol. 2018 Jul 24;18:181. doi: 10.1186/s12886-018-0812-4 (PMC6057085; doi:10.1186/s12886-018-0812-4)
Supplement: Supplementary file 3 — Ophthalmic centers. (DOCX 18 kb) [file 12886_2018_812_MOESM3_ESM.docx]

**Additional file 3**

| No. | Ophthalmic Center | Number of Patients supposed to be recruited |
| --- | --- | --- |
| 1 | Shanghai General Hospital | 1000 |
| 2 | Peking University People’s Hospital | 500 |
| 3 | Zhongshan Ophthalmic Center，Sun Yat-sen University | 500 |
| 4 | Tianjin Medical University Eye Hospital | 500 |
| 5 | Xijing Hospital | 500 |
| 6 | Qingdao Eye Hospital | 100 |
| 7 | Henan Eye Hospital | 100 |
| 8 | Shandong Eye Hospital | 100 |
| 9 | Eye and ENT Hospital of Fudan University | 100 |
| 10 | The First Affiliated Hospital of Anhui Medical University | 100 |
| 11 | Eye Hospital，Wenzhou Medical University | 100 |
| 12 | The Second Affiliated Hospital Of Nanchang University | 50 |
| 13 | Beijing Friendship Hospital, Capital Medical University | 50 |
| 14 | Xinhua Hospital Affiliated to Shanghai Jiao Tong University School of Medicine | 50 |
| 15 | Ruijin Hospital,Shanghai Jiao Tong University School of Medicine | 50 |
| 16 | Shanghai Tenth Hospital | 50 |
| 17 | Zhongshan Hospital of Fudan University | 50 |
| 18 | Ningbo First People’s Hospital | 50 |
| 19 | Zhangjiagang First People’s Hospital | 50 |
| 20 | Tianjin Eye Hospital | 50 |
| 21 | Peking Union Medical College Hospital | 50 |
| 22 | The First Affiliated Hospital of Guangxi University | 50 |
| 23 | The Second Affiliated Hospital of Soochow University | 50 |
| 24 | West China Hospital, Sichuan University | 50 |
| 25 | The First Affiliated Hospital of Xinjiang University | 50 |
| 26 | The Second Xiangya Hospital of Central South University | 50 |
| 27 | Ningxia Eye Hospital | 50 |
| 28 | Qilu Hospital of Shandong University | 50 |
| 29 | Yuncheng Eye Hospital | 50 |
| 30 | Lanzhou University Second Hospital | 50 |
| 31 | Eye Hospital, Nanjing Medical University | 50 |
| 32 | The Central Hospital of Lishui | 50 |
| 33 | Jinhua Municipal Central Hospital | 50 |
| 34 | Zhangjiang First People’s Hospital | 50 |
| 35 | The Second Affiliated Hospital of Dalian Medical University | 50 |
| 36 | The Second Affiliated Hospital of Xi’an Medical College | 50 |
| 37 | Xi’an No.3 Hospital | 50 |
| 38 | Xuzhou No.1 People’s Hospital | 50 |
| 39 | Xi’an No.1 Hospital | 50 |
| 40 | Ningbo Eye Hospital | 50 |
| 41 | Chaoju Ophthalmic Hospital | 50 |
| 42 | Qiqihar No.1 Hospital | 50 |
| 43 | The Forth Affiliated Hospital of China Medical University | 50 |
